# Cigarette smoking across life from 1946 to 2018: Harmonisation of four British birth cohort studies

**DOI:** 10.1111/add.70204

**Published:** 2025-10-02

**Authors:** Liam Wright, Loren Kock, Harry Tattan‐Birch, David Bann

**Affiliations:** ^1^ Centre for Longitudinal Studies, Social Research Institute University College London London UK; ^2^ Department of Behavioural Science and Health University College London London UK

**Keywords:** cohort effects, cross‐cohort analysis, life course analysis, smoking, tobacco, trends

## Abstract

**Background and Aims:**

Tobacco smoking has declined dramatically in many high‐income countries over the past seventy years. Studies that have mapped this trend have relied on repeat cross‐sectional or retrospectively measured smoking data, which have limitations regarding accurate measurement, inclusion of early smokers, and capturing of within‐person change over time. Here, we introduce a new resource detailing harmonisable smoking data in four British birth cohort studies spanning 1946–2018 and use these data to document age and cohort changes in smoking.

**Design:**

Longitudinal data from four nationally representative British Birth Cohort Studies, born 1946, 1958, 1970 and 2000/02, respectively.

**Setting:**

Great Britain.

**Participants:**

50 942 participants were eligible for inclusion in this study (5362 in the 1946c, 16 178 in the 1958c, 16 036 in the 1970c, and 13 366 in the 2001c). Data collection spanned the years 1946–2018.

**Measurements:**

Prevalence of daily smoking and cigarettes smoked per day were measured prospectively at various points across the life course via self‐report.

**Findings:**

The prevalence of smoking and the average number of cigarettes smoked by daily smokers declined between each successive cohort. At age 42/43y, prevalence of daily smoking was 33.6% (95% confidence interval [CI] = 31.8%, 35.5%) in the 1946c, 27.3% (95% CI = 26.5%, 28.2%) in the 1958c, and 22.1% (95% CI = 21.3%, 22.8%) in the 1970c. Males smoked more and with greater intensity than females, on average, though sex differences were smaller in latter cohorts. Within a cohort, the prevalence and intensity of smoking peaked in early adulthood (< age 30y) and declined thereafter; participants who continued to smoke daily smoked fewer cigarettes as they grew older.

**Conclusions:**

In Great Britain, smoking prevalence and cigarette consumption appear to have declined substantially between cohorts born across the latter half of the twentieth and early twenty‐first centuries. The British Birth Cohorts represent a unique and largely underutilized resource for investigating trends in smoking across life (prenatal to old age) and by year of birth (1946–2001), including changes in the determinants, correlates, and consequences of smoking. We provide syntax and information on items on smoking in these cohorts to catalyse future research, also available at: https://cls-data.github.io/smoking-in-the-cohorts/.

## INTRODUCTION

The British Birth Cohort Studies have been described as ‘jewels in the crown’ of United Kingdom (UK) social science [1]. These studies, which follow nationally representative samples of individuals born in 1946, 1958, 1970 and 2000/2002, respectively, offer a unique window into the major social changes that have occurred in Great Britain since the Second World War, such as the expansion of higher education, growing female labour force participation, changes in social mobility and increasing obesity rates [2, 3, 4, 5]. Beyond enabling researchers to document the size and timing of these trends, these cohorts allow investigation into changes in the determinants—and consequences—of socio‐economic and health outcomes, both between generations and over the life course [6, 7, 8, 9].

One health behaviour that has undergone substantial change over the past 70 years is cigarette smoking. The prevalence of smoking, which reached 80% among men in Great Britain in 1950 [10], declined dramatically following the discovery of tobacco's role in the aetiology of lung cancer and subsequent implementation of taxes, bans and other measures intended to curtail its use [11, 12]. Approximately 12% of UK adults now smoke [13, 14], one of the lowest rates in Western Europe [15], although smoking remains a leading cause of preventable death and social inequalities in health [16, 17, 18].

Cross‐sectionally, younger adults are more likely to smoke cigarettes as initiation typically occurs during adolescence or early adulthood, with more individuals quitting than taking up smoking thereafter [12, 14]. Yet, these age differences belie large cohort effects: at a given age, individuals born more recently are much less likely to have been a regular smoker [12, 19, 20, 21]. The precise pattern differs by sex [12, 20]: smoking prevalence among women peaked later than among men (although at a lower level) and exhibited a slower decline over time [12].

Existing studies on cross‐cohort changes in cigarette consumption have used repeat cross‐sectional data to create pseudo‐cohorts or have relied on (often older) people reporting their smoking history retrospectively [19, 20, 21, 22, 23, 24]. In contrast, smoking in each of the British Birth Cohort Studies has been measured prospectively and on multiple occasions over the life course. This has several advantages over the other approaches. Importantly, prospective measurement avoids survivor and recall biases and tracking the same individuals across time allows individual trajectories to be examined and complex behaviours (e.g. relapse or changes in smoking intensity) to be defined. Some early smokers will not appear in retrospectively measured or repeat cross‐sectional data because of premature mortality and earlier smoking may alternatively be misremembered or not reported, including—feasibly—if smoking is stigmatized, which is increasingly the case [25]. Further, repeated cross‐sectional data, in particular, cannot distinguish between changes in smoking being because of differences in uptake, cessation or premature mortality [21] and cannot clarify whether changes in smoking intensity are because of within person changes in consumption or differences in quit rates according to amount smoked, limiting the inferences that can be drawn.

Beyond avoiding these problems, birth cohort studies expand the range of research questions that can be asked. Most saliently, they enable investigation into life course determinants and consequences of smoking—for instance, on associations between maternal smoking and adult health [26]. The British Birth Cohort Studies, in particular, represent a rich seam of information for researchers to use. Each contains thousands of participants and tens of thousands of variables and include links with administrative data, biological assessments and genetic assays, much of which can be harmonized across the studies [27, 28] and used to perform appropriately powered analyses of individual trajectories and cross‐cohort change, including for important sub‐groups such as gender and socio‐economic groups [6].

In this study, we present the output of an effort to collate and harmonise data on smoking behaviour from the four British Birth Cohort Studies. We use this data to examine cohort and age‐related changes in several measures of tobacco consumption—including current and former daily smoking and smoking intensity (cigarettes per day)—overall and separately by gender, leveraging prospective and repeated measurement of participants. We reasoned that increased knowledge of smoking's harms and the implementation of measures (e.g. taxes) to curtail tobacco's use would mean that individuals have become less likely to start smoking, more likely to quit, and more likely to reduce the number of cigarettes they consume either to reduce health risks or lessen the financial cost of smoking (e.g. by drawing more nicotine from each cigarette) [29]. Therefore, our hypotheses (which were not prespecified in a protocol) were that: population prevalence of daily or ever smoking and the average number cigarettes smoked by smokers would be progressively lower between cohorts, and within a cohort, the prevalence of daily smoking among the total population and the number of cigarettes smoked daily by ‘sustained’ smokers would be highest in early adulthood (~age 25 years), declining thereafter.

Accompanying this paper is code to generate the harmonized variables so that researchers can use the data in their own analyses. We also provide detailed information on the smoking‐related data available in each cohort as a reference source (see Supporting information and osf.io/54w6q).

## METHODS

### Participants

The Medical Research Council (MRC) National Survey of Health and Development (hereafter, 1946c) follows a sample of individuals born in mainland Britain (England, Scotland or Wales) during a single week of March 1946. Participants were recruited by sampling singleton births, with individuals from households with non‐manual employment oversampled. The National Child Development Study (hereafter, 1958c) and the 1970 British Cohort Study (hereafter, 1970c) track all individuals born in mainland Britain in single weeks of March 1958 and April 1970, respectively. Immigrants to the United Kingdom born in these same weeks were later added using school enrolment information. The Millennium Cohort Study (hereafter, 2001c) follows a sample of individuals from across the United Kingdom born between 2000/2002. Participants were recruited using a two‐stage stratified sampling design with participants sampled from selected postcode areas. Individuals from Northern Ireland, Scotland and Wales, ethnic minority backgrounds, or disadvantaged areas were oversampled. Given differences in cohort eligibility and increasing ethnic diversity within the United Kingdom, to increase comparability, we restricted our analysis to singletons of White ethnicity born in England, Scotland or Wales. (Approximately, 1%, 5%, and 22% of the 1958c, 1970c and 2001c cohort members were non‐White; the 1946c are understood to all be of White ethnicity).

Each cohort and survey sweep has received ethical approval and obtained appropriate consents according to guidance in place at data collection. Further details on each study is available in cohort profiles [29, 30, 31, 32, 33, 34].

### Cigarette smoking

Data on smoking has been collected at various points over participants' life courses, including during adolescence for the 1958c, 1970c and 2001c, as well as on participants themselves and their family members (e.g. parents) and, more recently, on e‐cigarette use. The ages at which follow‐up sweeps have been performed differs across studies, but there is overlap between cohorts at several ages, including age 42/43 years when the 1946c, 1958c and 1970c were each interviewed (1989, 2000/2001 and 2012, respectively). A full description of the smoking data available in each cohort is provided in the Supporting information (as the cohorts are ongoing, a live version of the document is also available at https://osf.io/54w6q/). Here, we use variables for current daily smoking (participants and participants' mothers and fathers), smoking intensity (cigarettes per day; participants only), ever and former daily smoking (participants only) and daily smoking during pregnancy (participants' mothers). Data on smoking intensity was not available in the 2001c and data on smoking during pregnancy was not available in the 1946c. Additionally, parental smoking during cohort member's childhood was collected retrospectively (rather than prospectively) at age 53 years in the 1946c.

### Statistical analysis

We calculated descriptive statistics for each outcome, separately for each cohort sweep, for males and females combined and stratified by sex. For binary variables (e.g. current daily smoking), we calculated the proportion reporting the behaviour. For smoking intensity, we calculated the mean and SD for (a) the total population; (b) daily smokers (at a given sweep); and (c) sets of ‘sustained’ smokers who reported daily smoking at consecutive sweeps (e.g. at each sweep between age 20 years to 43 years in the 1946c). Focusing on changes in smoking intensity among sets of sustained smokers allowed us to account for compositional issues whereby individuals with the greatest nicotine dependence may quit less, and therefore, make up a progressively larger share of smokers over time.

As each cohort was subject to attrition, to address the loss of power and potential bias caused by sample loss [35], we used multiple imputation (MI) to impute missing values (m = 40, iterations = 10). Data were inputted in wide format so that earlier and later measurements of smoking behaviour could be used as predictors in imputation models. Observations were removed post‐imputation where the participant was known to be dead at that sweep. We used classification and regression trees (CART), a machine learning method, to account for potentially non‐linearity and collinearity in the relationship between variables. We included several earlier life predictors as auxiliary variables in imputation models: sex, participants' verbal cognitive ability at age 10/11 years, country of birth, parental socio‐economic position (family social class and mother's and father's education level), age, and parental height and body mass index (kg/m^2^). As a robustness check, we instead used inverse probability weights (IPW) to account for attrition with weights generated using these same auxiliary variables plus measurements of parental smoking, given these were collected in childhood; remaining item‐missingness for the variables in the response models was accounted for using MI.

Both weighting and MI assume that data are ‘missing at random’ (i.e. missingness is independent of the true value conditional on imputation model covariates). This may be incorrect if smokers are more likely to drop‐out of the study (although, note, earlier smoking observations are included in our imputation models). To explore the sensitivity of our results to selective attrition, we repeated (IPW) analyses for participants' prevalence of daily smoking assuming that smokers who dropped out of the study continued to smoke following the ‘Russell Standard’ [36].

We carried out all analyses using R version 4.3.1 [37]. Given the 1946c and 2001c used stratified study designs, we accounted for complex survey design using study‐specific probability (recruitment) weights. Our analysis plan was not pre‐registered—results should be considered exploratory.

## RESULTS

### Cohort members' cigarette smoking

**FIGURE 1:**
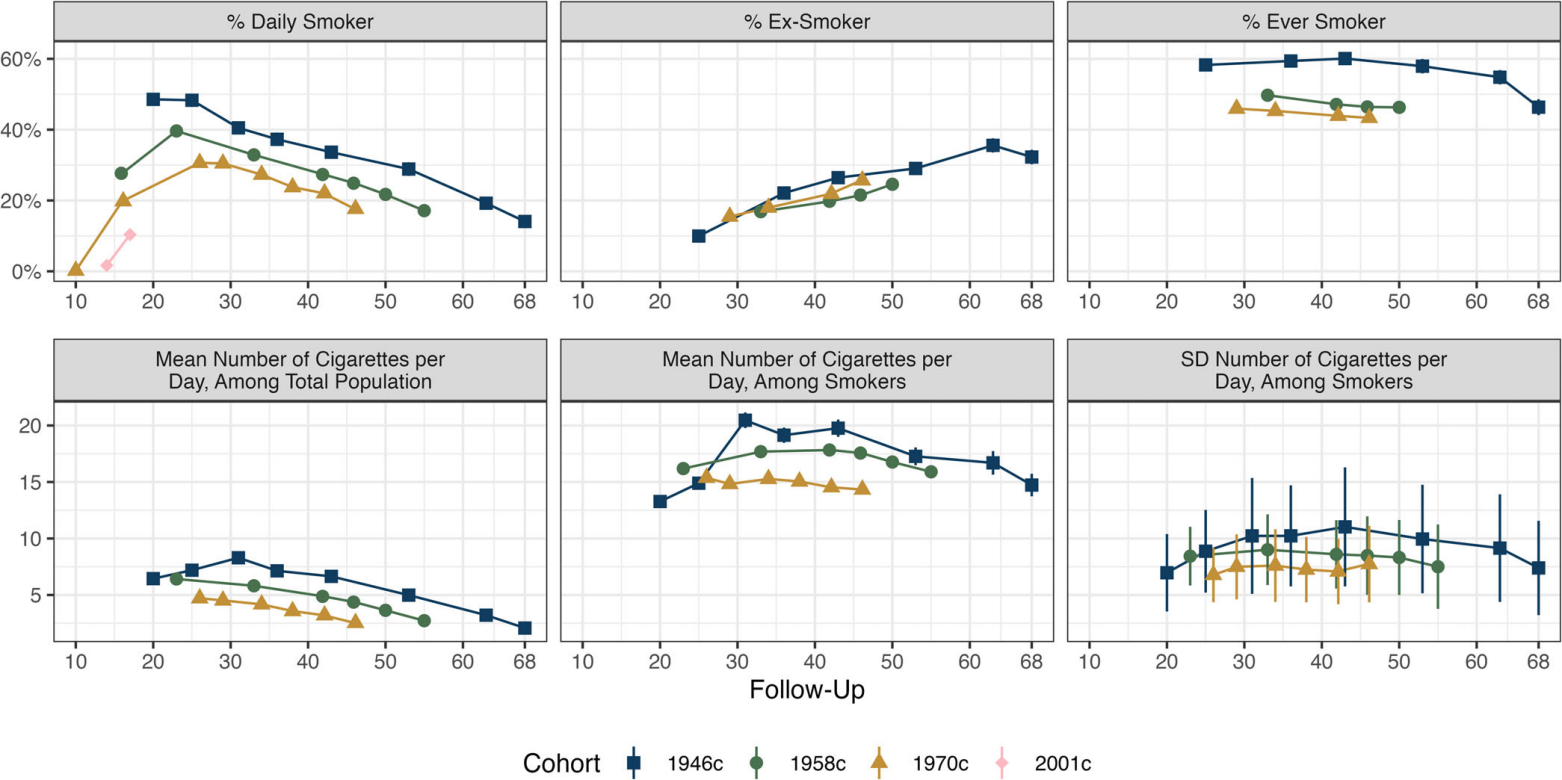
Cohort members' cigarette smoking, by cohort and age. Calculated using multiply imputed data (m = 40) and accounting for complex survey design.

There were 5362 eligible participants in the 1946c, 16 178 in the 1958c, 16 036 in the 1970c, and 13 366 in the 2001c. There were sizeable cross‐cohort differences in the proportion of participants who smoked daily (top left panel, Figure 1). At age 16/17 years, the prevalence of daily smoking was 27.7% (95% CI = 26.9%–28.5%) in the 1958c, 19.8% (95% CI = 18.9%–20.7%) in the 1970c and 10.4% (95% CI = 9.5%–11.2%) in the 2001c. Figures at age 42/43 years were 33.6% (95% CI = 31.8%–35.5%) in the 1946c, 27.3% (95% CI = 26.5%–28.2%) in the 1958c and 22.1% (95% CI = 21.3%–22.8%) in the 1970c. Prevalence increased during adolescence, reached its peak in early adulthood (ages 20–30 years) and declined thereafter. Age related declines during adulthood were similar in the 1946c, 1958c and 1970c.

The average number of cigarettes smoked daily across the total population also declined from early adulthood (bottom left panel, Figure 1). This largely reflected declining prevalence of daily smoking: the number of cigarettes smoked daily by daily smokers exhibited weaker change as participants aged (bottom middle panel, Figure 1). Nevertheless, there was evidence that, among sustained smokers (i.e. individuals who reported daily smoking in a contiguous run of sweeps), the average number of cigarettes smoked each day declined over time (Figure S1).

The average number of cigarettes smoked among smokers was successively lower between cohorts: at age 42/43 years, 19.8 (95% CI = 19.0–20.5) in the 1946c, 17.8 (95% CI = 17.5–18.1) in the 1958c and 14.5 (95% CI = 14.2–14.8) in the 1970c (bottom middle panel, Figure 1). There was also evidence of greater variation in earlier cohorts in the number of cigarettes smoked daily by smokers, although confidence intervals overlapped (bottom right panel, Figure 1).

### Sex‐specific trends in cohort members' cigarette smoking

**FIGURE 2:**
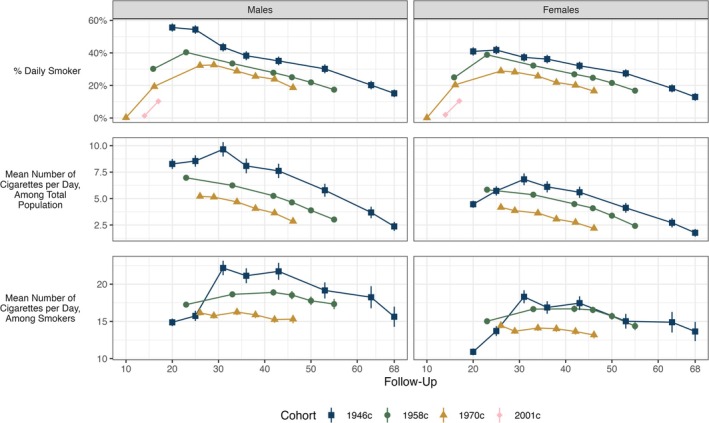
Cohort members' cigarette smoking, by cohort, sweep and sex. Calculated using multiply imputed data (m = 40) and accounting for complex survey design.

Men were generally more likely to be daily smokers than women, although these differences varied across time (top panels, Figure 2; see also Figures S1). Differences were particularly pronounced in early adulthood in the 1946c: at age 20 years, the prevalence of daily smoking was 40.9% (95% CI = 38.4%–43.5%) among females and 55.6% (95% CI = 53.1%–58.1%) among males. Differences between sexes later in adulthood in the 1946c and in the latter three cohorts were much smaller. At age 42/43 years, the difference in the prevalence of smoking in males compared with females was 3.0 percentage points (pp; 95% CI = −0.7 pp to 6.7 pp) in the 1946c and 0.9 pp (95% CI = −0.7 pp to 2.6 pp) in the 1958c and 3.7 pp (95% CI = 2.2 pp–5.2 pp) in the 1970c. The pattern of declining prevalence of smoking after age 30 years was observed in both sexes in each cohort (Figure 2; see also Figures S1 and S3).

The average number of cigarettes smoked daily was higher in men than women in each cohort, both measured across the total population (smokers and non‐smokers) and among daily smokers only (middle and bottom panels, Figure 2; see also Figures S4–S5). The difference in smoking intensity between male and female smokers was broadly stable across the life course, but somewhat small in later cohorts. Among daily smokers at age 42/43 years in the 1946c, average number of cigarettes smoked per day by male smokers was 4.3 cigarettes higher (95% CI = 2.8–5.7) than among female smokers. Corresponding figures were 2.2 (95% CI = 1.6–2.8) in the 1958c and 1.6 (95% CI = 1.0–2.2) in the 1970c.

### Parental cigarette smoking

**FIGURE 3:**
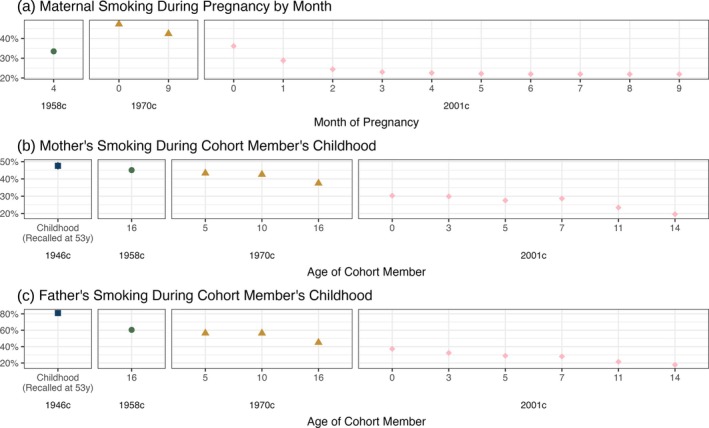
Cohort members' parents cigarette smoking, by cohort, sweep and sex. Calculated using multiply imputed data (m = 40) and accounting for complex survey design. Note different scales are used for the y‐axes.

Parents were born approximately 30 years before participants on average in each cohort. Maternal smoking during pregnancy was lower in the 2001c than 1958c or 1970c, but rates were higher in the 1970c than the 1958c (top panels, Figure 3). A total of 42.3% (95% CI = 41.5%, 43.1%) of participants' mothers smoked in month 9 of pregnancy in the 1970c, while 33.5% (95% CI = 32.8%–34.2%) of participants' mothers smoked in month 4 of pregnancy in the 1958c.

The prevalence of parental (daily) smoking during participants' childhood was highest in the 1946c and lowest in the 2001c: 80.5% (95% CI = 78.9%–82.2%) of fathers smoked in the 1946c, compared with 24.2% (95% CI = 22.8%–25.5%) at age 14 years in the 2001c (middle and bottom panels, Figure 3). Differences between 1958c and 1970c were smaller, especially for mother's smoking. When participants were age 16 years, 45.2% (95% CI = 44.3%–46.1%) of mother's smoked in the 1958c and 38.1% (95% CI = 37.2%–39.0%) in the 1970c.

### Sensitivity analyses

Results were qualitatively similar when using IPW instead of MI to account for attrition (Figure S6). However, in each cohort, smokers had a higher rate of attrition than non‐smokers, including at early ages before smoking‐related deaths may be expected to occur (Figure S7). Although we included smoking observations from earlier and later sweeps in imputation models, data could still be missing not at random (MNAR). Nevertheless, qualitatively similar results were obtained (albeit weaker in size) when adopting the ‘Russell Standard’ and assuming continual smoking for smokers who dropped out—there was lower prevalence of smoking in more recent cohorts and declining prevalence of smoking after early adulthood (Figure S8).

## DISCUSSION

We present new harmonised data for smoking behaviour across four British Birth Cohort Studies born 1946 to 2001. We used these resources to investigate long‐run changes in smoking by age and cohort. In each cohort with adulthood data, daily smoking peaked in early adulthood (~age 25 years) and declined thereafter (up to 69 years). At a given age, fewer people in more recent cohorts smoked, and those who did, smoked less per day. The prevalence of smoking among participants' parents was also lower in more recent cohorts. Differences were particularly large among participants' fathers—for instance, prevalence of smoking declined 55% between the 1946c and 2001c. We also observed sizable sex differences: males smoked more than females and the gender gap decreased between cohorts.

These results are consistent with previous analyses of UK data over shorter timespans (e.g. 1972–2019) that used pseudo‐cohorts or retrospective measurement of cigarette smoking [12, 19, 20, 21]. Our results extend these by identifying small changes on the intensive margin (i.e. how much to smoke), as well as on the extensive margin (i.e. whether to smoke at all). Among sustained smokers, average number of cigarettes smoked per day declined as individuals aged, although changes were weaker than changes in the total number of smokers.

As in other studies documenting smoking trends, multiple explanations are possible. For example, declines could reflect changes in beliefs about the health risks of smoking or be largely the consequence of policies designed to reduce tobacco use (e.g. the imposition of taxes). The period studied overlaps with substantial increases in the price of a packet of cigarettes (almost three‐fold increase in real terms since 1987) [38, 39], bans on smoking in indoor public places, public health messaging appearing on packing and changes to the minimum legal age of smoking [40]. However, we observed largely linear declines in smoking across adulthood, suggesting no single event led to such declines. We also cannot determine whether reductions in cigarette smoking translated into lower ingestion of tar or nicotine—smokers could have responded to higher prices by smoking each cigarette more deeply [41].

This analysis only scratches the surface of questions that can be asked with these cohort data, including questions on individual smoking trajectories and cross‐cohort differences. Rich socio‐economic data (e.g. social class, education and income) enable investigation of changing inequalities in smoking between cohorts and over the life course; detailed health and function data could be used for investigating cohort effects in smoking's association with morbidity and mortality outcomes; and genetic data [28] could be used to investigate changes in the genetic contribution to smoking at different stages of the tobacco epidemic [42] or before and after specific policy changes, for instance, as a test of the ‘hardening hypothesis' [11]. Future sweeps [43, 44, 45] may also be useful for investigating changes in vaping, which has become more widespread in recent years [46].

### Strengths and limitations

Strengths include the longitudinal, prospective measurement of cigarette smoking from a nationally representative sample of participants and their parents. Such data mitigate recall biases and allow tracking of smoking behaviour across life. Although parental smoking data was collected retrospectively from participants in the 1946c, estimates for fathers' smoking (~80%) were very similar to a contemporary data source [10].

Limitations include—common with all longitudinal studies—attrition. However, the rich birth cohort data enabled us to draw on early life predictors of attrition and use MI and IPW. Smokers were more likely to drop‐out in earlier sweeps. Nevertheless, our results were similar to those identified in repeated cross‐sectional data [21], which suffer from differing patterns of missing data (selection rather than attrition) and were qualitatively similar when adopting the very conservative Russell Standard assumption. Although the wording of questionnaire items was broadly semantically similar, exact wording often differed, both between cohorts and within a cohort across time, which could have influenced responses. However, our results matched our expectations and were consistent with prior work using other data sources. Although longitudinal measurement allowed us to assess within‐person change in smoking behaviour, we were unable to separate age and period (year) effects—though it is noteworthy that a peak in smoking in early adulthood was observed in each cohort for which data were available. Finally, to increase comparability between cohorts, we only examined those of White ethnicity. Given the ethnic diversity of the United Kingdom has increased over time, our cross‐cohort estimates do not capture differences resulting from this change in the composition of the population.

## AUTHOR CONTRIBUTIONS

All authors contributed to the analysis plan. Liam Wright performed the coding, analysis and data harmonisation and wrote the first draft of the manuscript. All authors provided critical revisions.

## DECLARATION OF INTERESTS

None.

## Supporting information


**Table S1.** Variables used to define current daily smoking by cohort‐sweep.
**Table S2.** Variables used to define current smoking intensity (cigarettes per day) by cohort‐sweep.
**Table S3.** Variables used to define former daily smoking by cohort‐sweep.
**Table S4.** Variables used to define parental smoking by cohort‐sweep.
**Figure S1.** Average number of cigarettes smoked per day among sustained smokers. Each line represents a set of sustained smokers defined by reporting smoking daily at each sweep along the line (e.g., for the 1946c, the line spanning 20y‐68y represents smokers reporting smoking at each sweep between and including 20y – 68y). Calculated using multiply imputed data (m = 40) and accounting for complex survey design.
**Figure S2.** Sex‐differences (male vs female) in cohort members' smoking behaviour, by cohort and sweep. Values above zero represent greater propensity of males to engage in the behaviour. Calculated using multiply imputed data (m = 40) and accounting for complex survey design.
**Figure S3.** Percentage of cohort members who smoke daily, by cohort, sweep, and sex. Calculated using multiply imputed data (m = 40) and accounting for complex survey design.
**Figure S4.** Mean number of cigarettes smoked per day among total sample by cohort, sweep, and sex. Calculated using multiply imputed data (m = 40) and accounting for complex survey design.
**Figure S5.** Mean number of cigarettes smoked per day among daily smokers by cohort, sweep, and sex. Calculated using multiply imputed data (m = 40) and accounting for complex survey design.
**Figure S6.** Cohort member smoking behaviour by cohort, sweep and estimator used. Left panel: multiple imputation. Middle panels: weighted for non‐response. Right panels: complete case data, not weighted for non‐response. All figures account for complex survey design.
**Figure S7.** Difference in probability of not responding in a given sweep (indicated by line colour) among smokers vs. non‐smokers by cohort and age at which smoking behaviour measured. Values above zero indicate smokers were less likely to respond at the sweep indicated by line coluor.
**Figure S8.** Estimated percentage of cohort members who smoke daily by cohort, sweep and whether previous daily smoking behaviour values were fed forward. Left panel: observed data (no feeding forward). Middle panel: feeding forward for smokers only (i.e., assumed to still be a smoker if missing and previous observed value was daily smoker). Right panel: feeding forward for all individuals (i.e., assumed to still be previous observed smoking status if missing for current sweep). All figures weighted for non‐response and account for complex survey design.
**Figure S9.** Data collection on smoking behaviour in the four British birth cohort studies and Next Steps. Blue shaded areas show age ranges in which (contemporary) smoking behaviour was collected in 3 or more cohorts.

## Data Availability

The code used to run the analysis is available at https://osf.io/54w6q/. Data from the National Child Development Study, British Cohort Study and Millennium Cohort Study are available through the UK Data Service (https://ukdataservice.ac.uk/). Data from the MRC National Survey of Health and Development are available via application on UCL Skylark (https://skylark.ucl.ac.uk/).
